# Assessment of the Quality of Life of Patients with Diabetes and Prediabetes in Poland: A Cross-Sectional Study

**DOI:** 10.3390/jcm14061883

**Published:** 2025-03-11

**Authors:** Mariola Mroz, Dorota Sadowska, Mateusz Zarychta, Grazyna Iwanowicz-Palus, Adam Kretowski, Mateusz Cybulski

**Affiliations:** 1 Department of Development in Midwifery, Faculty of Health Sciences, Medical University of Lublin, 20-081 Lublin, Poland; 2 Department of Endocrinology, Diabetology and Internal Medicine, Medical University of Bialystok, 15-276 Bialystok, Poland; 3 Faculty of Technical Physics, Information Technology and Applied Mathematics, Lodz University of Technology, 93-005 Lodz, Poland; 4 Department of Integrated Medical Care, Faculty of Health Sciences, Medical University of Bialystok, 15-096 Bialystok, Poland

**Keywords:** diabetes, prediabetes, quality of life (QoL), the Medical Outcomes Study 36-Item Short-Form Health Survey (SF-36)

## Abstract

**Background**: Diabetes mellitus is one of the greatest public health challenges worldwide and one of the major conditions contributing to a poorer quality of life. The main factors that may deteriorate quality of life among patients with diabetes include age, financial status, educational background, quality of healthcare services and presence of disease complications. This study aimed to assess the quality of life among patients with type 1 and type 2 diabetes and prediabetes in Poland using the example of the Podlaskie Province, taking into account selected sociodemographic variables. **Methods**: A total of 874 patients participated in the study, including 448 women (55.8%) and 386 men (44.2%). The study was conducted from July 2022 to July 2023 among participants of the “Zatrzymaj cukrzycę! Polski Rejestr Diabetologiczny PolReD” (“Stop Diabetes! Polish Diabetes Registry (PolRed)”) project or those hospitalised in the Department of Endocrinology, Diabetology and Internal Medicine at the University Clinical Hospital in Bialystok. The study used a diagnostic survey method using a survey questionnaire developed by the authors and the 36-Item Short Form Survey (SF-36). **Results**: The overall study group had the highest level of quality of life assessment in the domains of social functioning (M = 69.48; SD ± 28.07), physical functioning (M = 64.54; SD ± 31.57) and role limitations due to emotional problems (M = 62.40; SD ± 45.21), and the lowest level of quality of life in the domain of general health perceptions (M = 42.21; SD ± 12.77). Age was found to be negatively correlated in all quality of life domains analysed (r = −0.438; *p* < 0.001)—quality of life decreased with age in all investigated domains. Men had a statistically significantly (*p* < 0.05) higher quality of life in each analysed domain (M = 43.52–74.08; SD ± 12.68–44.09) compared to women (M = 41.18–65.88; SD ± 12.76–46.08). Place of residence also exhibited a statistically significant (*p* < 0.05) differentiated quality of life in terms of physical functioning. **Conclusions**: The assessment of quality of life among patients with type 1 and type 2 diabetes and those diagnosed with prediabetes from the Podlaskie Province depends on the type of hyperglycaemic disorder. The assessment of quality of life among patients with type 1 and type 2 diabetes and prediabetes is determined by specific socio-demographic factors, including, above all, age and gender. Respondents with type 1 diabetes have a higher quality of life in terms of role limitations due to physical health, role limitations due to emotional problems, pain (bodily pain) and general health compared to respondents with type 2 diabetes.

## 1. Background

Diabetes mellitus is a metabolic disease that involves abnormally high levels of glucose in the blood. The major subtypes of diabetes are type 1 diabetes and type 2 diabetes, which classically result from defective insulin secretion (type 1 diabetes) and/or action (type 2 diabetes). Type 1 diabetes occurs in children or adolescents, while type 2 diabetes is thought to affect middle-aged and older adults who have prolonged hyperglycaemia due to poor lifestyle and dietary choices. The pathogenesis of two types of diabetes is drastically different, so each type has a different aetiology, symptoms and treatment [1]. Regardless of the specific type of diabetes, complications include microvascular, macrovascular and neurovascular problems. Microvascular and macrovascular complications vary with the degree and duration of poorly controlled diabetes and include nephropathy, retinopathy, neuropathy and atherosclerotic cardiovascular disease events, especially if associated with other comorbidities such as dyslipidaemia and hypertension [2].

Diabetes is one of the greatest public health challenges worldwide [3]. According to the International Diabetes Federation, nearly 537 million people worldwide had diabetes in 2021, including 61 million in Europe. This figure is expected to rise to more than 783 million by 2045 [3]. Based on projections from the NCD Risk Factor Collaboration (NCD-RisC), as many as 828 million adults (people aged 18 years or older) had diabetes in 2022, an increase of 630 million compared to 1990 [4]. According to the International Diabetes Federation, 2.67 million people in Poland will have diabetes in 2021 [3].

Prediabetes is a condition characterised by elevated blood glucose levels, but below the threshold for diagnosing diabetes, which is associated with a higher risk of developing diabetes in the future [5]. Prediabetes is diagnosed based on laboratory measurement of fasting blood glucose (FBG), glycated haemoglobin (HbA1c) or 2-h post-load blood glucose (2hBG) [6,7]. The definition of prediabetes is used to identify people who are at risk of developing diabetes in the future, but prediabetes is also associated with a high burden of cardiometabolic risk factors [8]. The increasing prevalence of prediabetes worldwide is a serious public health problem and, when combined with the growing epidemic of diabetes and its complications, is not encouraging [7]. The global prevalence of impaired glucose tolerance (IGT) in 2021 was 9.1% (464 million) and is projected to increase to 10.0% (638 million) in 2045. The global prevalence of impaired fasting glucose (IFG) in 2021 was 5.8% (298 million) and is projected to increase to 6.5% (414 million) in 2045. The prevalence of IGT and IFG in 2021 was highest in high-income countries. In 2045, the largest relative increase in IGT and IFG cases is projected to occur in low-income countries [5].

Quality of life (QoL) is a significant element of people’s lives and is connected to the culture and value systems in which they live, as well as their objectives and requirements [9]. In general, people with diseases tend to have a poorer quality of life than healthy people. It is complicated to enhance people’s physical well-being excluding quality of life, especially for those diagnosed with chronic non-communicable diseases [10]. The World Health Organisation has identified diabetes as one of the major non-communicable diseases and one of the highest risk factors for premature mortality around the world. This is why diabetes mellitus is one of the major conditions contributing to a poorer quality of life [11,12]. The main factors that may deteriorate quality of life among patients with diabetes include age [13], financial status [14], educational background [15], quality of healthcare services [16] and presence of disease complications [17]. A poorer quality of life leads to negative consequences involving both the physical health and mental health of individuals with diabetes [18].

The conducted study has the potential to fill several important gaps in the literature, in particular: (1) Comparison of the quality of life of people with diabetes and prediabetes, because in the literature, especially in Poland, there are few studies directly comparing the quality of life of people with diabetes and people with prediabetes; (2) Differentiation of results depending on the type of diabetes—studies on the quality of life of people with diabetes often focus on patients with type 1 or type 2 diabetes, but there are no analyses comparing the quality of life of patients with both types of the disease, and, even more so, studies that additionally take into account people diagnosed with prediabetes.

In light of the above, this study aimed to evaluate the quality of life of patients with type 1 and type 2 diabetes and prediabetes in Poland as exemplified by the Podlaskie Province, taking into account selected sociodemographic variables such as age, gender, place of residence and BMI value. Quality of life varies according to the type of diabetes diagnosed and two specific research hypotheses were put forward: (1) Quality of life in patients with diabetes and those diagnosed with prediabetes is determined by the indicated socio-demographic factors. (2) The diagnosis of diabetes type and prediabetes differentiates quality of life.

## 2. Materials and Methods

### 2.1. Cross-Sectional Design

The study was conducted between July 2022 and July 2023. The Head of the Department of Endocrinology, Diabetology and Internal Medicine of the University Clinical Hospital in Bialystok and the Clinical Research Centre of the Medical University of Bialystok gave his consent to conduct the study.

The selection of the sample in this study was purposeful and non-probabilistic, due to the subject of the research. The people taking part in the study met its established criteria, which could not be met in the case of random selection.

The inclusion criteria for the study were

- age above 18 years,
- diagnosis of type 1 diabetes or diagnosis of type 2 diabetes, or diagnosis of prediabetes,
- no psycho-physical disorders (persons able to reliably describe their subjective feelings and to fill in the questionnaire form independently).

The exclusion criteria for the study were

- lack of consent to participate in the study,
- age below 18 years,
- no diagnosis of type 1 diabetes, type 2 diabetes or prediabetes,
- existing psycho-physical disorders.

The study covered 936 people, of which 874 complete and correctly completed questionnaires were obtained, which were qualified for further statistical analysis. The success rate of the data obtained was 93.38%. Questionnaires with incomplete data or incorrectly completed sheets were not further analysed.

### 2.2. Study Group

The study included 874 patients participating in the project “Zatrzymaj Cukrzycę! Polski Rejestr Diabetologiczny (PolRed)” (“Stop Diabetes! Polish Diabetes Registry (PolRed)”) project or hospitalised in the Department of Endocrinology, Diabetology and Internal Medicine of the University Clinical Hospital in Bialystok, including 448 women (55.8%) and 386 men (44.2%).

### 2.3. Measures and Data Collection

The research was carried out by employing a diagnostic survey using the questionnaire technique and by means of a document study using the document analysis technique. Medical records of patients of the Department of Endocrinology, Diabetology and Internal Medicine of the University Clinical Hospital in Bialystok and the Clinical Research Centre of the Medical University of Bialystok were used for the analysis.

The research tool consisted of two components:

- The survey questionnaire developed by the authors taking into account the characteristics of the respondents (sociodemographic variables).
- The Medical Outcomes Study 36-Item Short-Form Health Survey, which is designed for self-assessment of health-related quality of life. It consists of 11 questions with 36 statements to identify 8 categories of quality of life, i.e., physical functioning, role limitations due to physical health, role limitations due to emotional problems, energy/fatigue, emotional well-being, social functioning, pain (bodily pain/physical pain), general health. The quality of life index is the sum of the scores of all eight subscales. It is assumed that the sum of the scores in an indicator shows its positive or negative value, which means that a differently configured scoring can be used depending on whether the level of positive assessments about health or the level of negative assessments is of interest. The rule of thumb is that the higher the score, the more positive the respondent’s self-assessment in terms of the proposed aspects of quality of life. According to the original version of the questionnaire, the highest point value indicates the highest assessment of quality of life, while the lowest point value indicates the lowest assessment of quality of life. The value of Cronbach’s alpha coefficient for the individual subscales is 0.63–0.95 [19,20,21].

### 2.4. Procedure and Ethical Considerations

The study was conducted following the recommendations and was reviewed and approved by the Bioethics Committee of the Medical University in Bialystok (statute no. APK.002.264.2022 of 23 June 2022). All participants gave written informed consent in accordance with the Declaration of Helsinki [22]. Respondents were informed of the anonymity and voluntariness of their participation in the study and that the collected data obtained would be used for research objectives only. Consent was obtained from patients to participate in the study after discussing the study design and purpose with them.

### 2.5. Statistical Analysis

The collected research material was statistically processed using the statistical package IBM SPSS Statistics (ver. 21) (IBM, Armonk, NY, USA).

Quantitative variables were described by mean, standard deviation, quartiles, measures of symmetry and kurtosis, as well as minimum and maximum values. For qualitative variables, the count and percentage of each category were provided.

Appropriate statistical tests were applied to verify the hypotheses. The normality of data distributions was checked using the Shapiro–Wilk test. When the assumptions for parametric tests (variables measured at the quantitative level of measurement) were met, the independent samples Student’s *t*-test was used to verify the hypothesis of equality of means of the analysed variable in two populations, and the one-way analysis of variance (ANOVA) for independent groups was used to verify the hypothesis of equality of means of the analysed variable in several populations.

The statistical analyses carried out also used the Tukey test for comparing pairs of means as one of the post-hoc tests (multiple comparison procedures), performed after obtaining a significant F-value following an analysis of variance (ANOVA), showing means that differ in statistical significance, taking into account the number of degrees of freedom (df), i.e., the number of independent observational outcomes minus the number of relationships that link these outcomes to each other (the number of degrees of freedom can be equated with the number of independent random variables that influence the outcome).

To determine the correlation between quantitative variables, Pearson’s linear correlation coefficient was used, which is designed to test for a linear relationship between two characteristics, as long as the distribution of the analysed characteristics is normal [23].

To determine the strength of the correlation, a classification according to J.P. Guilford [24] was used:

- 0—no correlation,
- 0.0 < |r| ≤ 0.1—slight correlation,
- 0.1 < |r| ≤ 0.3—weak (low) correlation,
- 0.3 < |r| ≤ 0.5—average correlation,
- 0.5 < |r| ≤ 0.7—high correlation,
- 0.7 < |r| ≤ 0.9—very high correlation,
- 0.9 < |r| < 1.0—almost complete correlation,
- |r| = 1—complete correlation.

The results obtained were assumed to be statistically significant at the significance level of *p* < 0.05.

## 3. Results

The study comprised 874 subjects from the Podlaskie Province—patients of the Department of Endocrinology, Diabetology and Internal Medicine of the University Clinical Hospital in Bialystok and the Clinical Research Centre of the Medical University in Bialystok, including 124 patients with type 1 diabetes (14.2%), 581 patients with type 2 diabetes (66.5%) and 169 subjects with prediabetes (19.3%). Overall, women (55.8%), people aged 51–70 years (42.2%), residents of provincial cities (50.0%) and those with a BMI indicating obesity (44.2%) predominated in the study group. Detailed data are presented in Table 1.

Table 2 presents an analysis of the quality of life of the study participants overall, those with type 1 and type 2 diabetes and those diagnosed with prediabetes in terms of the individual domains of the SF-36 questionnaire. It has been shown that

- in the group of subjects with type 1 diabetes, the highest scores, signifying the highest assessment of quality of life, were recorded in the domains of physical functioning (M = 81.49; SD ± 24.14), social functioning (M = 75.50; SD ± 23.28), role limitations due to emotional problems (M = 73.66; SD ± 40.16), and the lowest score, signifying the lowest assessment of quality of life, in the domain of general health (M = 43.75; SD ± 13.59);
- in the group of subjects with type 2 diabetes, the highest scores, signifying the highest assessment of quality of life, were recorded in the domains of social functioning (M = 67.86; SD ± 29.49) and emotional well-being (M = 60.93; SD ± 17.49), and the lowest score, signifying the lowest assessment of quality of life, in the domains of general health (M = 41.31; SD ± 12.29) and pain (M = 49.60; SD ± 31.11);
- in the group of subjects diagnosed with prediabetes, the highest scores, signifying the highest assessment of quality of life, were recorded in the domains of physical functioning (M = 73.52; SD ± 26.00), social functioning (M = 70.64; SD ± 25.63), role limitations due to emotional problems (M = 65.09; SD ± 42.70), and the lowest score, signifying the lowest assessment of quality of life, in the domain of general health (M = 44.20; SD ± 13.49);
- the overall study group had the highest scores signifying the highest assessment of quality of life, in the domains of social functioning (M = 69.48; SD ± 28.07), physical functioning (M = 64.54; SD ± 31.57) and role limitations due to emotional problems (M = 62.40; SD ± 45.21), and the lowest score indicating the lowest level of quality of life, in the domain of general health (M = 42.21; SD ± 12.77).

**Table 2:** Analysis of the quality of life of study participants with type 1 and type 2 diabetes and diagnosed prediabetes by domain.

Quality of Life Domain	M	SD	A	K	Min	Max	Q_1_	Mdn	Q_3_
Type 1 diabetes									
Physical functioning	81.49	24.14	−1.79	2.61	0.00	100.00	75.00	90.00	100.00
Role limitations due to physical health	63.51	41.61	−0.58	−1.38	0.00	100.00	25.00	75.00	100.00
Role limitations due to emotional problems	73.66	40.16	−1.09	−0.57	0.00	100.00	41.67	100.00	100.00
Energy/fatigue	55.24	18.24	−0.10	0.30	0.00	100.00	45.00	55.00	65.00
Emotional well-being	60.93	16.06	−0.59	0.34	10.00	91.00	54.25	62.00	70.00
Social functioning	75.50	23.28	−0.90	0.56	0.00	100.00	62.50	75.00	100.00
Pain (bodily/physical pain)	62.56	29.12	−0.51	−0.67	0.00	100.00	45.00	67.50	90.00
General health	43.75	13.59	0.02	−0.34	15.00	80.00	35.00	45.00	55.00
Type 2 diabetes									
Physical functioning	58.31	32.51	−0.41	−1.10	0.00	100.00	30.00	65.00	85.00
Role limitations due to physical health	50.09	45.51	−0.02	−1.84	0.00	100.00	0.00	50.00	100.00
Role limitations due to emotional problems	59.21	46.55	−0.38	−1.76	0.00	100.00	0.00	100.00	100.00
Energy/fatigue	52.65	19.65	−0.13	0.11	0.00	100.00	40.00	50.00	65.00
Emotional well-being	60.93	17.49	−0.56	0.45	0.00	100.00	52.00	62.00	71.00
Social functioning	67.86	29.49	−0.63	−0.59	0.00	100.00	50.00	75.00	100.00
Pain (bodily/physical pain)	49.60	31.11	−0.04	−1.12	0.00	100.00	22.50	50.00	77.50
General health	41.31	12.29	0.07	−0.33	5.00	75.00	30.00	40.00	50.00
Prediabetes									
Physical functioning	73.52	26.00	−1.07	0.45	0.00	100.00	60.00	80.00	95.00
Role limitations due to physical health	55.18	42.07	−0.29	−1.62	0.00	100.00	0.00	75.00	100.00
Role limitations due to emotional problems	65.09	42.70	−0.65	−1.35	0.00	100.00	0.00	100.00	100.00
Energy/fatigue	54.67	17.36	−0.38	0.29	0.00	90.00	45.00	55.00	65.00
Emotional well-being	61.63	16.88	−0.67	0.48	8.00	91.00	52.00	62.00	73.00
Social functioning	70.64	25.63	−0.70	−0.21	0.00	100.00	62.50	75.00	100.00
Pain (bodily/physical pain)	55.10	30.07	−0.15	−0.99	0.00	100.00	35.00	57.50	80.00
General health	44.20	13.49	0.19	−0.09	5.00	80.00	35.00	40.00	55.00
Total									
Physical functioning	64.54	31.57	−0.67	−0.77	0.00	100.00	40.00	75.00	90.00
Role limitations due to physical health	52.97	44.53	−0.15	−1.78	0.00	100.00	0.00	75.00	100.00
Role limitations due to emotional problems	62.40	45.21	−0.52	−1.60	0.00	100.00	0.00	100.00	100.00
Energy/fatigue	53.41	19.04	−0.18	0.17	0.00	100.00	40.00	55.00	65.00
Emotional well-being	61.06	17.16	−0.58	0.44	0.00	100.00	52.00	62.00	72.00
Social functioning	69.48	28.07	−0.70	−0.38	0.00	100.00	50.00	75.00	100.00
Pain (bodily/physical pain)	52.51	30.95	−0.13	−1.08	0.00	100.00	25.00	57.50	77.50
General health	42.21	12.77	0.11	−0.24	5.00	80.00	35.00	40.00	50.00

Abbreviations: M—arithmetic mean, SD—standard deviation, A—asymmetry, K—kurtosis, Min—minimum, Max—maximum, Q_1_—lower quartile, Mdn—median, Q_3_—upper quartile.

Statistical analysis showed differences between subjects with type 1 diabetes, type 2 diabetes and prediabetes in physical functioning (*p* < 0.001), role limitations due to physical health (*p* < 0.05), role limitations due to emotional problems (*p* < 0.05), pain—physical pain (*p* < 0.001) and general health (*p* < 0.05).

Post-hoc tests showed that, for physical functioning, all study groups differed statistically significantly (*p* < 0.001). The highest scores were found in the group with type 1 diabetes (M = 81.49; SD ± 24.14), and the lowest among patients with type 2 diabetes (M = 58.31; SD ± 26.00); thus, higher quality of life in terms of physical functioning was demonstrated by study participants with type 1 diabetes compared to those with type 2 diabetes. Subjects diagnosed with prediabetes showed statistically significantly higher scores in the physical functioning domain (M = 73.52; SD ± 26.00) compared to subjects with type 2 diabetes (M = 73.52; SD ± 26.00). On the other hand, higher scores were reported in the group with type 1 diabetes (M = 81.49; SD ± 24.14) compared with subjects diagnosed with prediabetes (M = 73.52; SD ± 26.00).

For the quality of life domains of role limitations due to physical health, role limitations due to emotional problems, pain—physical pain and general health, post-hoc tests showed statistically significant (*p* < 0.05) differences between subjects with type 1 diabetes and those with type 2 diabetes.

Respondents with type 1 diabetes had a higher quality of life (role limitations due to physical health—M = 63.51; SD ± 41.61; role limitations due to emotional problems—M = 73.66; SD ± 59.21; pain—M = 62.56; SD ± 49.60; general health—M = 43.75; SD ± 14.31), compared to subjects with type 2 diabetes (role limitations due to physical health—M = 50.09; SD ± 45.51; role limitations due to emotional problems—M = 59.21; SD ± 46.50; pain—M = 49.60; SD ± 31.11; general health—M = 41.31; SD ± 12.29). Details are presented in Table 3.

The total study group showed negative correlations of age in all analysed domains of quality of life (r = −0.438; *p* < 0.001)—quality of life decreased with age in all analysed domains. Subjects with type 1 diabetes showed negative correlations of age with physical functioning (r = −0.437; *p* < 0.001), pain—physical pain (r = −0.178; *p* < 0.05) and general health (r = −0.225; *p* < 0.05); quality of life decreased with age in domains including physical functioning, pain and general health. The group of subjects with type 2 diabetes showed negative correlations of age in all domains of quality of life (r = −0.082—r = −0.509; *p* < 0.05); quality of life decreased with age in all domains analysed. The prediabetes group showed negative correlations of age with physical functioning (r = −0.425; *p* < 0.001), role limitations due to physical health (r = −0.275; *p* < 0.001), role limitations due to emotional problems (r = −0.235; *p* < 0.01), pain—physical pain (r = −0.235; *p* < 0.01) and general health (r = −0.186; *p* < 0.05). This means that, in this group of subjects, quality of life in the domains of physical functioning, role limitations due to physical health, role limitations due to emotional problems, pain—physical pain and general health decreased with age (Table 4).

In the total study group, men had a statistically significant (*p* < 0.05) higher quality of life in each analysed domain (M = 43.52–74.08; SD ± 12.68–44.09) compared to women (M = 41.18–65.88; SD ± 12.76–46.08). In the group of subjects with type 1 diabetes, there were no statistically significant (*p* > 0.05) differences between women and men. In the group of subjects with type 2 diabetes, men had a statistically significant (*p* < 0.05) higher quality of life in each analysed domain (M = 43.13–73.27; SD ± 12.43–44.90) compared to women (M = 39.76–63.26; SD ± 11.98–47.35). In the group of subjects diagnosed with prediabetes, men had a statistically significant (*p* < 0.05) higher quality of life in the domains of energy/fatigue (M = 60.34; SD ± 14.72) and pain (M = 63.66; SD ± 29.66) compared to women (energy/fatigue M = 51.74; SD ± 17.96; pain M = 50.63; SD ± 29.43, respectively) (Table 5).

In the total study group, place of residence statistically significantly (*p* < 0.05) differentiated quality of life in terms of physical functioning. Post hoc tests showed a difference between residents of provincial cities and rural areas in terms of physical functioning. Rural residents had a statistically significant (*p* < 0.05) higher quality of life in terms of physical functioning (M = 70.07; SD ± 28.15) compared to residents of provincial cities (M = 61.96; SD ± 32.98). In the group of subjects with type 1 diabetes and type 2 diabetes, there were no statistically significant (*p* > 0.05) differences between residents of provincial cities, residents of cities/towns other than provincial and residents of rural areas. In the group of subjects diagnosed with prediabetes, residents of provincial cities had a statistically significant (*p* < 0.05) higher quality of life in the domain of general health (M = 46.25; SD ± 23.56) compared to residents of cities/towns other than provincial ones (M = 41.92; SD ± 12.97) (Table 6).

The total group of subjects showed negative correlations of BMI in quality of life domains of physical functioning (r = −0.155; *p* < 0.001), pain complaints (r = −0.088; *p* < 0.01) and general health (r = −0.119; *p* < 0.001); the higher the subjects’ BMI, the lower their quality of life in physical functioning, pain—physical pain and general health. In the group of subjects with type 1 diabetes, BMI was negatively correlated with physical functioning (r = −0.367; *p* < 0.001); the higher the BMI of the subjects, the lower the quality of life in terms of physical functioning. The group of subjects with type 2 diabetes showed negative correlations of BMI with general health (r = −0.126; *p* < 0.01); the higher the BMI, the lower the quality of life in terms of general health. The group of subjects diagnosed with prediabetes showed negative correlations of BMI in quality of life domains of physical functioning (r = −0.216; *p* < 0.01), role limitation due to physical health (r = −0.156; *p* < 0.05) and pain—physical pain (r = −0.088; *p* < 0.01); the higher the subjects’ BMI, the lower their quality of life in terms of physical functioning, role limitation due to physical health and pain (Table 7).

## 4. Discussion

Diabetes mellitus is a disease that interferes with many spheres of a person’s life, affecting their everyday functioning in family, professional and social areas. It thus determines the patient’s quality of life in specific domains. Quality of life is a very broad concept, reflecting satisfaction and well-being, and is a measure of goal achievement, satisfaction and self-acceptance. Maintaining or improving quality of life, in addition to the medical goals of treatment, is a crucial part of the therapeutic process nowadays [25].

Among the patients with glycaemic disorders participating in this study, the highest scores in the assessment of quality of life were recorded in the domains of social functioning (M = 69.47), physical functioning (M = 64.54) and role limitations due to emotional problems (M = 62.40), and the lowest scores in the domain concerning their perceptions of general health (M = 42.21). Furthermore, in the assessment of quality of life, the study groups differed in statistical significance (*p* < 0.001). The highest scores in the domain of physical functioning were found in the group with type 1 diabetes (M = 81.49), while the lowest scores were found among people with type 2 diabetes (M = 58.31). In this domain, individuals diagnosed with prediabetes showed statistically significantly higher scores (M = 73.52) compared to subjects with type 2 diabetes (M = 58.31). Furthermore, those with type 1 diabetes had a higher quality of life in terms of role limitations due to physical health (*p* = 0.012) and emotional problems (*p* = 0.009), pain (*p* < 0.001) and general health (*p* = 0.024), compared to those with type 2 diabetes. Badura-Brzoza et al. [26], in their comparative analysis of a group of patients with type 1 and type 2 diabetes, reported significantly worse physical functioning scores among patients with type 2 diabetes (*p* = 0.0012). However, they found no differences between the study groups in the other domains of quality of life. The results of the study by Burkiewicz et al. (2017) [27] show that patients with type 2 diabetes rated their quality of life as good (62%). Opinions on quality of life were influenced by health assessment, awareness of the disease and emotional factors such as feeling safe and accepting the disease. A better perception of health was also associated with a higher quality of life for respondents surveyed by Majda et al. (2013) [28]. An analysis by Trybusinska and Matusiak (2021) [29] showed that diabetes patients most often rated their quality of life as good (57%) and were also satisfied with their health (44.9%). They functioned best in the environmental and physical domains and slightly worse in the social relations domain and the psychological domain. Pazderska (2017) [30] obtained similar findings on the quality of life as assessed by patients with diabetes.

Sociodemographic factors may influence diabetic patients’ assessment of their quality of life. Namdeo et al. (2023) [31] and Majda et al. (2013) [28] showed that the quality of life of type 1 and type 2 diabetes patients decreases with age. The analysis of those authors’ study corresponds with the results of the present study, showing that the lower the age of the diabetes patients, the better their quality of life in all domains analysed (*p* < 0.001). Age significantly influenced the assessment of quality of life among patients with type 1 and type 2 diabetes and those with prediabetes. In a study by Trybusinska and Matusiak [29], it was observed that the older the patients were, the (slightly) lower they assessed their quality of life in the physical (*p* < 0.0001), psychological (*p* < 0.02) and social relations (*p* < 0.02) domains.

Available research reports indicate a correlation between gender and quality of life in individuals with diabetes; patients with higher scores in quality of life are men [28,29,31,32]. The results of the analyses conducted in this study confirm this correlation. Men participating in the study, compared to women, were characterised by a statistically significant higher quality of life in all domains (*p* < 0.05). Similar correlations were noted in the group of participants with type 2 diabetes, while only in the domains of energy/fatigue and pain among those diagnosed with prediabetes.

Place of residence also influences the perceived quality of life among diabetes patients. Analysis of the results of this study showed that rural area residents (M = 70.07), compared to those living in a provincial city (M = 61.96) had a statistically significant (*p* = 0.036) higher quality of life in terms of physical functioning. In the group of subjects diagnosed with prediabetes, those living in a provincial city had a higher quality of life in the domain of general health (*p* = 0.035). Namdeo et al. (2023) [31] also found a correlation between the place of residence and the level of quality of life of patients with diabetes—those living in cities had higher quality of life scores. On the other hand, in a study by Trybusinska and Matusiak (2021) [29], a trend was observed that, the larger the population of the agglomeration in which the respondents resided, the more their quality of life decreased in the physical, social and environmental domains, but no significant relationship was found for the analysed variable (*p* > 0.05).

In recent years, the dynamic development of new technologies and methods of treating diabetes has also significantly affected the quality of life of this group of patients. An example are new drugs that lower glucose levels, because modern therapies can significantly affect compliance with treatment and the well-being of patients, which is tantamount to improving the quality of life. A promising progress in the development of diabetology is, for example, the development of insulin taken once a week, because the reduced frequency of dosing of this drug can improve compliance with insulin therapy, reduce the burden of treatment and potentially improve the overall quality of life [33]. Similarly, glucagon-like peptide 1 (GLP-1) analogues have become an important part of the treatment of type 2 diabetes in the last decade. In addition to playing a key role in regulating blood glucose levels, GLP-1 analogues have been shown to reduce body weight and improve cardiovascular function, making them a valuable tool in the treatment of type 2 diabetes. Clinical studies indicate that these therapies result in better glucose control compared to traditional oral medications such as metformin and in combination with other therapies such as insulin [34]. Thus, patients who experience stabilised glucose levels are more likely to report improved quality of life, with less stress associated with the need to constantly monitor glucose levels and a lower risk of diabetes-related complications. One of the key effects of using GLP-1 analogues is their impact on weight loss. Patients with type 2 diabetes often struggle with obesity, which further worsens the course of the disease. GLP-1 analogue reduces appetite and improves metabolism, which leads to weight loss [35]. GLP-1 analogue may also have a beneficial effect on the mental health of patients. Studies show that patients who use GLP-1 analogues are less likely to complain about depression, compared to those who do not use this therapy [36]. These effects may result from improved glycaemic control, weight loss and overall improved health, which has a beneficial effect on patient well-being.

Continuous glucose monitoring (CGM) has also become an important tool in diabetes management. CGM allows for continuous, real-time monitoring of glucose levels, which allows patients to more precisely adjust their therapy and make decisions in response to changes in glycaemia. Continuous glucose monitoring allows for more precise tracking of glucose fluctuations throughout the day and night, which allows for better glycaemic control compared to traditional glucose monitoring with a glucometer. The ability to respond immediately to changes in glucose levels helps avoid hypoglycaemia or hyperglycaemia, which has a direct impact on the well-being and health of patients. Studies have shown that the use of CGM in patients with type 1 diabetes significantly reduces the time spent in hypoglycaemia and improves glycaemic control [37]. Better glucose control reduces the risk of diabetes complications such as retinopathy, neuropathy and nephropathy, which has a positive impact on long-term quality of life. Using CGM can also reduce anxiety and stress associated with self-monitoring, as patients receive more detailed information about their glucose levels in real time, which can increase their sense of security. Studies have shown that CGM reduces anxiety associated with hypoglycaemia and improves patients’ overall well-being [38]. Patients who use this technology often report feeling less out of control over their disease, which can lead to better mental health and greater motivation to adhere to treatment. Studies show that patients who use CGM are more likely to make healthy eating decisions and engage in regular physical activity, which improves their quality of life [39]. In addition, the ability to automatically adjust insulin doses to alter glucose levels in the body can improve the effectiveness of therapy and reduce the need to make difficult decisions in crisis situations. Although continuous glucose monitoring gives patients more control over their disease, it can also introduce some restrictions in everyday life. It requires patients to constantly wear the device, which can be uncomfortable, especially for those who prefer more discreet disease management. On the other hand, technologies such as CGM can also improve the quality of social life, allowing patients to be more confident in social and professional situations because it reduces the unpredictability of disease management [40].

### Limitations

The study is not without its limitations. First, it was a cross-sectional study. A cross-sectional study is useful for assessing quality of life at one point in time because it allows for collecting data at a specific point in time, which can be helpful in assessing the overall health, well-being and perception of quality of life in a given population. However, cross-sectional studies have some important limitations, especially in the context of assessing causality, and, in particular, the inability to determine the direction of causality. In cross-sectional studies, it is not possible to determine what is the cause and what is the effect; the lack of tracking changes over time: a cross-sectional study collects data at one point in time, which limits the ability to analyse how factors may change or how they influence each other over a long period. Caution is needed when interpreting results; due to the lack of a temporal context, results can be misleading in terms of cause and effect. Correlations can only be described, but conclusions about causes cannot be drawn. In summary, cross-sectional studies are useful for obtaining a picture of the situation at a given point in time, but they do not allow for drawing conclusions about the causes of changes in quality of life. For a deeper understanding of cause–effect relationships, a longitudinal study would be more appropriate. Second, it was a cross-sectional study based primarily on a self-report questionnaire. Although the scales used are sensitive instruments, they all focus on subjective feelings rather than objective criteria, creating a risk of false positives. Third, the study was conducted for a single province and not the whole of Poland, though this does not detract from the fact that the group was representative in terms of size in relation to the entire country.

## 5. Conclusions

Quality of life assessment in patients with type 1 and type 2 diabetes and diagnosed prediabetes from the Podlaskie Province depend on the type of hyperglycaemic disorder. The assessment of quality of life among patients with type 1 and type 2 diabetes and prediabetes is determined by specific socio-demographic factors, including, above all, age and gender. Respondents with type 1 diabetes have a higher quality of life in terms of role limitations due to emotional problems, role limitations due to emotional problems, pain (physical pain) and general health compared to respondents with type 2 diabetes.

## Figures and Tables

**Table 1:** Sociodemographic characteristics of respondents (n = 874).

Variable		Type 1 Diabetes (n = 124; 14.2%)		Type 2 Diabetes (n = 581; 66.5%)		Prediabetes (n = 169; 19.3%)		Total (n = 874; 100%)	
		n	%	n	%	n	%	n	%
Age (years)	below 50	96	77.4	93	16.0	79	46.7	268	30.7
	51–70	27	21.8	281	48.4	61	36.1	369	42.2
	over 70	1	0.8	207	35.6	29	17.2	237	27.1
Gender	female	63	50.8	314	54.0	111	65.7	448	55.8
	male	61	49.2	267	46.0	58	34.3	386	44.2
Place of residence	provincial city	60	48.4	299	51.5	78	46.2	437	50.0
	city/town other than provincial	53	42.7	233	40.1	79	46.7	365	41.8
	rural area	11	8.9	49	8.4	12	7.1	72	8.2
BMI	underweight	5	4.0	4	0.7	1	0.6	10	1.2
	normal	53	42.7	115	20.0	47	28.0	215	24.8
	overweight	37	29.8	171	29.7	51	30.4	259	29.8
	obesity	29	23.4	286	49.7	69	41.1	384	44.2

**Table 3:** Analysis of the correlation between domain-specific quality of life and diagnosis of type 1 and type 2 diabetes and prediabetes.

		M	SD	F	df	*p*	Tukey Test
Physical functioning	type 1 (I) diabetes	81.49	24.14	77.653	2	<0.001	I-II; II-III; I-III
	type 2 (II) diabetes	58.31	32.51				
	prediabetes (III)	73.52	26.00				
Role limitations due to physical health	type 1 (I) diabetes	63.51	41.61	8.808	2	0.012	I-II
	type 2 (II) diabetes	50.09	45.51				
	prediabetes (III)	55.18	42.07				
Role limitations due to emotional problems	type 1 (I) diabetes	73.66	40.16	9.483	2	0.009	I-II
	type 2 (II) diabetes	59.21	46.55				
	prediabetes (III)	65.09	42.70				
Energy/fatigue	type 1 (I) diabetes	55.24	18.24	3.150	2	0.207	-
	type 2 (II) diabetes	52.65	19.65				
	prediabetes (III)	54.67	17.36				
Emotional well-being	type 1 (I) diabetes	60.93	16.06	0.260	2	0.878	-
	type 2 (II) diabetes	60.93	17.49				
	prediabetes (III)	61.63	16.88				
Social functioning	type 1 (I) diabetes	75.50	23.28	5.175	2	0.075	-
	type 2 (II) diabetes	67.86	29.49				
	prediabetes (III)	70.64	25.63				
Pain (bodily/physical pain)	type 1 (I) diabetes	62.56	29.12	18.990	2	<0.001	I-II
	type 2 (II) diabetes	49.60	31.11				
	prediabetes (III)	55.10	30.07				
General health	type 1 (I) diabetes	43.75	13.59	7.434	2	0.024	I-II
	type 2 (II) diabetes	41.31	12.29				
	prediabetes (III)	44.20	13.49				

Abbreviations: M—arithmetic mean, SD—standard deviation, F—analysis of variance (ANOVA), df—number of degrees of freedom, *p*—test probability.

**Table 4:** Correlation analysis of age of study participants with type 1 and type 2 diabetes and diagnosed prediabetes with quality of life by domain.

Age								
Quality of Life Domain	Type 1 Diabetes		Type 2 Diabetes		Prediabetes		Total Study Group	
	r	*p*	r	*p*	r	*p*	r	*p*
Physical functioning	−0.438 **	<0.001	−0.509 ***	<0.001	−0.425 **	<0.001	−0.542 ***	<0.001
Role limitations due to physical health	−0.095	0.294	−0.328 **	<0.001	−0.275 *	<0.001	−0.301 **	<0.001
Role limitations due to emotional problems	−0.127	0.159	−0.414 **	<0.001	−0.235 *	0.002	−0.352 **	<0.001
Energy/fatigue	0.087	0.335	−0.143 *	0.001	0.043	0.578	−0.096	<0.001
Emotional well-being	0.068	0.454	−0.082 *	0.047	−0.017	0.827	−0.047	<0.001
Social functioning	−0.073	0.422	−0.220 *	<0.001	−0.091	0.237	−0.202 *	<0.001
Pain (bodily/physical pain)	−0.178 *	0.048	−0.272 *	<0.001	−0.235 *	0.002	−0.290 *	<0.001
General health	−0.225 *	0.012	−0.241 *	<0.001	−0.186 *	0.015	−0.239 *	<0.001

Abbreviations: r—Pearson’s correlation coefficient, *p*—test probability, *—weak (low) correlation, **—average correlation, ***—high correlation.

**Table 5:** Analysis of quality of life domains according to the gender of the study participants with respect to type of diabetes and prediabetes.

Quality of Life Domain	Gender	M	SD	t	df	*p*
Type 1 diabetes						
Physical functioning	male	79.67	26.39	−0.825	122	0.411
	female	83.25	21.80			
Role limitations due to physical health	male	63.52	41.48	0.004	122	0.997
	female	63.49	42.08			
Role limitations due to emotional problems	male	76.50	39.13	0.775	122	0.440
	female	70.90	41.26			
Energy/fatigue	male	56.31	19.21	0.641	122	0.523
	female	54.21	17.35			
Emotional well-being	male	63.10	16.00	1.489	122	0.139
	female	58.83	15.96			
Social functioning	male	77.46	24.24	0.919	122	0.360
	female	73.61	22.35			
Pain (bodily/physical pain)	male	66.27	27.40	1.401	122	0.164
	female	58.97	30.49			
General health	male	42.62	13.98	−0.908	122	0.366
	female	44.84	13.23			
Type 2 diabetes						
Physical functioning	male	65.75	30.86	5.196	579	<0.001
	female	51.99	32.58			
Role limitations due to physical health	male	59.18	44.90	4.513	579	<0.001
	female	42.36	44.66			
Role limitations due to emotional problems	male	67.79	44.15	4.179	574	<0.001
	female	51.91	47.35			
Energy/fatigue	male	56.31	19.50	4.199	579	<0.001
	female	49.54	19.27			
Emotional well-being	male	64.23	17.10	4.254	579	<0.001
	female	58.12	17.36			
Social functioning	male	73.27	27.84	4.134	579	<0.001
	female	63.26	30.11			
Pain (bodily/physical pain)	male	56.49	29.97	5.021	579	<0.001
	female	43.75	30.91			
General health	male	43.13	12.43	3.307	557	0.001
	female	39.76	11.98			
Prediabetes						
Physical functioning	male	73.28	28.62	−0.088	167	0.930
	female	73.65	24.66			
Role limitations due to physical health	male	57.76	43.48	0.575	167	0.566
	female	53.83	41.45			
Role limitations due to emotional problems	male	63.22	43.57	−0.411	167	0.682
	female	66.07	42.40			
Energy/fatigue	male	60.34	14.72	3.351	137	0.001
	female	51.71	17.96			
Emotional well-being	male	64.72	14.51	1.735	167	0.085
	female	60.01	17.84			
Social functioning	male	73.92	24.48	1.206	167	0.229
	female	68.92	26.16			
Pain (bodily/physical pain)	male	63.66	29.66	2.726	167	0.007
	female	50.63	29.43			
General health	male	46.29	12.23	1.462	167	0.146
	female	43.11	14.03			
Total						
Physical functioning	male	69.08	30.27	3.809	872	<0.001
	female	60.95	32.14			
Role limitations due to physical health	male	59.65	44.09	3.975	872	<0.001
	female	47.69	44.21			
Role limitations due to emotional problems	male	68.48	43.37	3.588	846	<0.001
	female	57.58	46.08			
Energy/fatigue	male	56.92	18.82	4.906	872	<0.001
	female	50.64	18.77			
Emotional well-being	male	64.12	16.53	4.746	872	<0.001
	female	58.64	17.28			
Social functioning	male	74.03	26.80	4.304	872	<0.001
	female	65.88	28.55			
Pain (bodily/physical pain)	male	59.11	29.73	5.715	872	<0.001
	female	47.28	30.92			
General health	male	43.52	12.68	2.705	872	0.007
	female	41.18	12.76			

Abbreviations: M—arithmetic mean, SD—standard deviation, t—independent samples Student’s *t*-test, df—number of degrees of freedom, *p*—test probability.

**Table 6:** Analysis of quality of life domains according to the place of residence of the study participants, taking into account the type of diabetes and prediabetes.

Quality of Life Domain	Place of Residence	M	SD	F	df	*p*	Tukey Test
Type 1 diabetes							
Physical functioning	provincial city (I)	81.83	23.47	0.145	2;121	0.865	**-**
	city/town other than provincial (II)	81.89	23.27				
	rural area (III)	77.73	32.89				
Role limitations due to physical health	provincial city (I)	61.25	42.04	0.194	2;121	0.824	-
	city/town other than provincial (II)	65.09	41.99				
	rural area (III)	68.18	40.45				
Role limitations due to emotional problems	provincial city (I)	75.00	40.07	0.634	2;121	0.532	-
	city/town other than provincial (II)	74.84	39.17				
	rural area (III)	60.61	46.71				
Energy/fatigue	provincial city (I)	55.17	19.79	0.001	2;121	0.999	-
	city/town other than provincial (II)	55.28	16.80				
	rural area (III)	55.45	17.81				
Emotional well-being	provincial city (I)	60.42	17.50	0.228	2;121	0.796	-
	city/town other than provincial (II)	61.94	14.08				
	rural area (III)	58.82	17.97				
Social functioning	provincial city (I)	75.42	26.44	0.005	2;121	0.995	-
	city/town other than provincial (II)	75.71	18.90				
	rural area (III)	75.00	26.22				
Pain (bodily/physical pain)	provincial city (I)	64.00	28.23	0.322	2;121	0.725	-
	city/town other than provincial (II)	62.22	29.81				
	rural area (III)	56.36	32.45				
General health	provincial city (I)	42.33	13.10	0.652	2;121	0.523	-
	city/town other than provincial (II)	44.91	13.78				
	rural area (III)	45.91	15.78				
Type 2 diabetes							
Physical functioning	provincial city (I)	55.52	33.93	2.900	2;166	0.056	**-**
	city/town other than provincial (II)	60.30	31.08				
	rural area (III)	65.92	28.75				
Role limitations due to physical health	provincial city (I)	49.83	45.84	0.609	2;166	0.544	-
	city/town other than provincial (II)	51.72	45.22				
	rural area (III)	43.88	45.22				
Role limitations due to emotional problems	provincial city (I)	57.97	47.52	0.217	2;166	0.805	-
	city/town other than provincial (II)	60.52	45.23				
	rural area (III)	60.54	47.47				
Energy/fatigue	provincial city (I)	51.99	20.73	1.454	2;166	0.234	-
	city/town other than provincial (II)	52.55	18.74				
	rural area (III)	57.14	16.65				
Emotional well-being	provincial city (I)	61.35	17.34	1.276	2;166	0.280	-
	city/town other than provincial (II)	59.77	17.81				
	rural area (III)	63.84	16.75				
Social functioning	provincial city (I)	67.43	29.67	0.075	2;166	0.928	-
	city/town other than provincial (II)	68.19	28.78				
	rural area (III)	68.88	32.19				
Pain (bodily/physical pain)	provincial city (I)	49.55	32.80	0.003	2;166	0.997	-
	city/town other than provincial (II)	49.71	29.46				
	rural area (III)	49.44	28.64				
General health	provincial city (I)	40.97	12.53	0.510	2;166	0.601	-
	city/town other than provincial (II)	41.42	12.08				
	rural area (III)	42.86	11.90				
Prediabetes							
Physical functioning	provincial city (I)	71.35	26.89	0.722	2;168	0.487	-
	city/town other than provincial (II)	74.68	26.27				
	rural area (III)	80.00	16.79				
Role limitations due to physical health	provincial city (I)	73.52	26.00	0.638	2;168	0.529	-
	city/town other than provincial (II)	51.28	42.24				
	rural area (III)	58.86	42.56				
Role limitations due to emotional problems	provincial city (I)	56.25	38.62	2.075	2;168	0.129	-
	city/town other than provincial (II)	58.55	44.68				
	rural area (III)	72.15	40.45				
Energy/fatigue	provincial city (I)	61.11	39.78	1.266	2;168	0.285	-
	city/town other than provincial (II)	52.44	17.26				
	rural area (III)	56.84	17.62				
Emotional well-being	provincial city (I)	55.00	15.81	1.850	2;168	0.161	-
	city/town other than provincial (II)	59.28	17.11				
	rural area (III)	64.28	17.20				
Social functioning	provincial city (I)	59.42	10.33	1.038	2;168	0.356	-
	city/town other than provincial (II)	68.43	26.48				
	rural area (III)	73.58	25.55				
Pain (bodily/physical pain)	provincial city (I)	65.63	19.31	2.180	2;168	0.116	-
	city/town other than provincial (II)	51.47	29.39				
	rural area (III)	60.03	31.08				
General health	provincial city (I)	46.25	23.56	3.411	2;168	0.035	I-II
	city/town other than provincial (II)	41.92	12.97				
	rural area (III)	47.03	13.17				
Total							
Physical functioning	provincial city (I)	61.96	32.98	3.324	2;871	0.036	I-III
	city/town other than provincial (II)	66.55	30.24				
	rural area (III)	70.07	28.15				
Role limitations due to physical health	provincial city (I)	51.66	44.78	0.849	2;871	0.428	-
	city/town other than provincial (II)	55.21	44.36				
	rural area (III)	49.65	43.91				
Role limitations due to emotional problems	provincial city (I)	60.41	46.34	1.135	2;871	0.322	-
	city/town other than provincial (II)	65.11	43.72				
	rural area (III)	60.65	45.56				
Energy/fatigue	provincial city (I)	52.51	20.01	1.569	2;871	0.209	-
	city/town other than provincial (II)	53.88	18.28				
	rural area (III)	56.53	16.48				
Emotional well-being	provincial city (I)	60.86	17.30	0.229	2;871	0.796	-
	city/town other than provincial (II)	61.06	17.24				
	rural area (III)	62.33	16.02				
Social functioning	provincial city (I)	68.71	28.77	0.383	2;871	0.682	-
	city/town other than provincial (II)	70.45	26.99				
	rural area (III)	69.27	29.37				
Pain (bodily/physical pain)	provincial city (I)	51.88	31.93	0.632	2;871	0.532	-
	city/town other than provincial (II)	53.76	30.27				
	rural area (III)	49.97	28.25				
General health	provincial city (I)	41.33	12.67	2.121	2;871	0.121	-
	city/town other than provincial (II)	43.14	12.77				
	rural area (III)	42.92	13.18				

Abbreviations: M—arithmetic mean, SD—standard deviation, F—analysis of variance (ANOVA), df—number of degrees of freedom, *p*—test probability.

**Table 7:** Correlation analysis of the BMI of type 1 diabetes, type 2 diabetes and diagnosed prediabetes study participants with quality of life by domain.

BMI (Body Mass Index)								
Quality of Life Domain	Type 1 Diabetes		Type 2 Diabetes		Prediabetes		Total Study Group	
	r	*p*	r	*p*	r	*p*	r	*p*
Physical functioning	−0.367 **	<0.001	−0.047	0.261	−0.216 *	0.005	−0.155 *	<0.001
Role limitations due to physical health	−0.074	0.411	−0.008	0.845	−0.156 *	0.044	−0.064	0.059
Role limitations due to emotional problems	−0.031	0.731	−0.016	0.694	−0.037	0.631	−0.044	0.192
Energy/fatigue	0.000	0.997	−0.054	0.198	0.000	0.999	−0.047	0.170
Emotional well-being	0.155	0.086	0.008	0.851	−0.011	0.889	0.020	0.550
Social functioning	0.013	0.886	0.047	0.263	−0.037	0.630	0.007	0.839
Pain (bodily/physical pain)	−0.056	0.536	−0.021	0.618	−0.198 *	0.010	−0.088 *	0.009
General health	−0.041	0.655	−0.126 *	0.002	−0.092	0.237	−0.119 *	<0.001

Abbreviations: r—Pearson’s correlation coefficient, *p*—test probability, *—weak (low) correlation, **—average correlation.

## Data Availability

The raw data supporting the conclusions of this article will be made available by the authors on request.

## References

[1] Sapra A., Bhandari P. (2025). Diabetes. StatPearls.

[2] Yamazaki D., Hitomi H., Nishiyama A. (2018). Hypertension with diabetes mellitus complications. Hypertens. Res..

[3] Sun H., Saeedi P., Karuranga S., Pinkepank M., Ogurtsova K., Duncan B.B., Stein C., Basit A., Chan J.C.N., Mbanya J.C. (2022). IDF Diabetes Atlas: Global, regional and country-level diabetes prevalence estimates for 2021 and projections for 2045. Diabetes Res. Clin. Pract..

[4] NCD Risk Factor Collaboration (NCD-RisC) (2024). Worldwide trends in diabetes prevalence and treatment from 1990 to 2022: A pooled analysis of 1108 population-representative studies with 141 million participants. Lancet.

[5] Rooney M.R., Fang M., Ogurtsova K., Ozkan B., Echouffo-Tcheugui J.B., Boyko E.J., Magliano D.J., Selvin E. (2023). Global Prevalence of Prediabetes. Diabetes Care.

[6] American Diabetes Association (2025). 2. Diagnosis and Classification of Diabetes: Standards of Medical Care in Diabetes-2025. Diabetes Care.

[7] Echouffo-Tcheugui J.B., Selvin E. (2021). Prediabetes and What It Means: The Epidemiological Evidence. Annu. Rev. Public Health.

[8] American Diabetes Association (2025). 3. Prevention or Delay of Diabetes and Associated Comorbidities: Standards of Medical Care in Diabetes-2025. Diabetes Care.

[9] (1995). The World Health Organization Quality of Life assessment (WHOQOL): Position paper from the World Health Organization. Soc. Sci. Med..

[10] Tamornpark R., Utsaha S., Apidechkul T., Panklang D., Yeemard F., Srichan P. (2022). Quality of life and factors associated with a good quality of life among diabetes mellitus patients in northern Thailand. Health Qual. Life Outcomes.

[11] Reynolds R., Dennis S., Hasan I., Slewa J., Chen W., Tian D., Bobba S., Zwar N. (2018). A systematic review of chronic disease management interventions in primary care. BMC Fam. Pract..

[12] Krzemińska S., Bąk E., Polanská A., Hašová K., Laurinc M., Zrubcová D., Młynarska A. (2023). Does gender affect health-related quality of life in patients with type 2 diabetes (ADDQoL) in Central European countries?. Ann. Agric. Environ. Med..

[13] Zurita-Cruz J.N., Manuel-Apolinar L., Arellano-Flores M.L., Gutierrez-Gonzalez A., Najera-Ahumada A.G., Cisneros-González N. (2018). Health and quality of life outcomes impairment of quality of life in type 2 diabetes mellitus: A cross-sectional study. Health Qual. Life Outcomes.

[14] ElShazly H.M., Hegazy N.N. (2017). Socioeconomic determinants affecting the quality of life among diabetic and hypertensive patients in a rural area, Egypt. J. Family Med. Prim. Care.

[15] Nielsen H.B., Ovesen L.L., Mortensen L.H., Lau C.J., Joensen L.E. (2016). Type 1 diabetes, quality of life, occupational status and education level—A comparative population-based study. Diabetes Res. Clin. Pract..

[16] Teli M., Thato R., Rias Y.A. (2023). Predicting Factors of Health-Related Quality of Life Among Adults With Type 2 Diabetes: A Systematic Review. SAGE Open Nurs..

[17] Trikkalinou A., Papazafiropoulou A.K., Melidonis A. (2017). Type 2 diabetes and quality of life. World J. Diabetes.

[18] Degu H., Wondimagegnehu A., Yifru Y.M., Belachew A. (2019). Is health related quality of life influenced by diabetic neuropathic pain among type II diabetes mellitus patients in Ethiopia?. PLoS ONE.

[19] Ware J.E., Sherbourne C.D. (1992). The MOS 36-item short-form health survey (SF-36). I. Conceptual framework and item selection. Med. Care.

[20] Ware J.E., Snow K.K., Kosinski M., Gandek B. (1993). SF-36 Health Survey. Manual and Interpretation Guide.

[21] Żołnierczyk-Zreda D. (2010). The Polish version of the SF-36v2 questionnaire for the quality of life assessment. Przegl. Lek..

[22] World Medical Association (2013). World Medical Association Declaration of Helsinki: Ethical principles for medical research involving human subjects. JAMA.

[23] Schober P., Boer C., Schwarte L.A. (2018). Correlation Coefficients: Appropriate Use and Interpretation. Anesth. Analg..

[24] Guilford J.P. (1965). Fundamental Statistics in Psychology and Education.

[25] Korzon-Burakowska A., Adamska K., Skuratowicz-Kubica A., Jaworska M., Świerblewska E., Kunicka K. (2010). Effect of education level on diabetes control and quality of life in insulin-treated type 2 diabetic patients. Diabet. Prakt..

[26] Badura-Brzoza K., Główczyński P., Piegza M., Błachut M., Nabrdalik K., Gumprecht J., Gorczyca G. (2022). Comparative assessment of the relationship between emotional factors and quality of life in a group of patients with type 1 and type 2 diabetes—Preliminary report. Psychiatr. Pol..

[27] Burkiewicz A., Stasiuk J.J., Kozłowski D. (2017). Disease awareness and its impact on quality of life and cooperation with the medical team in patients with type II diabetes. Ann. Acad. Med. Gedan..

[28] Majda A., Walas K., Morawa J. (2013). The quality of life of persons with diabetes type 2 in health resort hospital. Probl. Pielęg..

[29] Trybusińska D., Matusiak G., Raczkowski A., Stencel M., Łukasiewicz J., Kowalski W., Ačinovič T.I. Quality of life in diabetic patients in the era of the SARS-COV-2 pandemic. The Spring of the Autumn Selected Aspects Concerning the Life Satisfaction of Elderly People.

[30] Pazderska M. (2017). The quality of life and dietetic conditioning of diabetes patients receiving outpatient treatment. Pielęgniarstwo w Opiece Długoterminowej.

[31] Namdeo M.K., Verma S., Gupta R., Islam R., Nazneen S., Rawal L. (2023). Depression and health-related quality of life of patients with type 2 diabetes attending tertiary level hospitals in Dhaka, Bangladesh. Glob. Health Res. Policy.

[32] Jakubowska E., Jakubowski K., Cipora E. (2010). Satisfaction with life among patients with diabetes. Probl. Hig. Epidemiol..

[33] Karakasis P., Patoulias D., Pamporis K., Popovic D.S., Stachteas P., Bougioukas K.I., Fragakis N., Rizzo M. (2023). Efficacy and safety of once-weekly versus once-daily basal insulin analogues in the treatment of type 2 diabetes mellitus: A systematic review and meta-analysis. Diabetes Obes. Metab..

[34] Marso S.P., Bain S.C., Consoli A., Eliaschewitz F.G., Jódar E., Leiter L.A., Lingvay I., Rosenstock J., Seufert J., Warren M.L. (2016). Semaglutide and Cardiovascular Outcomes in Patients with Type 2 Diabetes. N. Engl. J. Med..

[35] Popoviciu M.S., Păduraru L., Yahya G., Metwally K., Cavalu S. (2023). Emerging Role of GLP-1 Agonists in Obesity: A Comprehensive Review of Randomised Controlled Trials. Int. J. Mol. Sci..

[36] Onoviran O.F., Li D., Toombs Smith S., Raji M.A. (2019). Effects of glucagon-like peptide 1 receptor agonists on comorbidities in older patients with diabetes mellitus. Ther. Adv. Chronic Dis..

[37] Beck R.W., Riddlesworth T., Ruedy K., Ahmann A., Bergenstal R., Haller S., Kollman C., Kruger D., McGill J.B., Polonsky W. (2017). Effect of Continuous Glucose Monitoring on Glycemic Control in Adults With Type 1 Diabetes Using Insulin Injections: The DIAMOND Randomized Clinical Trial. JAMA.

[38] Kłak A., Mańczak M., Owoc J., Olszewski R. (2021). Impact of continuous glucose monitoring on improving emotional well-being among adults with type 1 diabetes mellitus: A systematic review and meta-analysis. Pol. Arch. Intern. Med..

[39] Schubert-Olesen O., Kröger J., Siegmund T., Thurm U., Halle M. (2022). Continuous Glucose Monitoring and Physical Activity. Int. J. Environ. Res. Public Health.

[40] Burge M.R., Mitchell S., Sawyer A., Schade D.S. (2008). Continuous Glucose Monitoring: The Future of Diabetes Management. Diabetes Spectr..

